# Malarial Pigment Hemozoin and the Innate Inflammatory Response

**DOI:** 10.3389/fimmu.2014.00025

**Published:** 2014-02-05

**Authors:** Martin Olivier, Kristin Van Den Ham, Marina Tiemi Shio, Fikregabrail Aberra Kassa, Sophie Fougeray

**Affiliations:** ^1^Department of Medicine, McGill TB International Centre, Research Institute of the McGill University Health Centre, McGill University, Montréal, QC, Canada; ^2^Department of Microbiology and Immunology, McGill TB International Centre, Research Institute of the McGill University Health Centre, McGill University, Montréal, QC, Canada

**Keywords:** malaria, hemozoin, macrophages, signaling, inflammasome

## Abstract

Malaria is a deadly infectious disease caused by the intraerythrocytic protozoan parasite *Plasmodium*. The four species of *Plasmodium* known to affect humans all produce an inorganic crystal called hemozoin (HZ) during the heme detoxification process. HZ is released from the food vacuole into circulation during erythrocyte lysis, while the released parasites further infect additional naive red blood cells. Once in circulation, HZ is rapidly taken up by circulating monocytes and tissue macrophages, inducing the production of pro-inflammatory mediators, such as interleukin-1β (IL-1β). Over the last few years, it has been reported that HZ, similar to uric acid crystals, asbestos, and silica, is able to trigger IL-1β production via the activation of the NOD-like receptor containing pyrin domain 3 (NLRP3) inflammasome complex. Additionally, recent findings have shown that host factors, such as fibrinogen, have the ability to adhere to free HZ and modify its capacity to activate host immune cells. Although much has been discovered regarding NLRP3 inflammasome induction, the mechanism through which this intracellular multimolecular complex is activated remains unclear. In the present review, the most recent discoveries regarding the capacity of HZ to trigger this innate immune complex as well as the impact of HZ on several other inflammatory signaling pathways will be discussed.

## Introduction

Malaria is an infection that affects 216 million individuals worldwide. Every year, approximately 700,000 people succumb to this devastating disease (1). The protozoan parasite *Plasmodium* is the etiological agent of malaria and it is transmitted during the blood meal of a female *Anopheles* mosquito (2). Of all the *Plasmodium* species infecting humans, *P. falciparum* is the most virulent and its pathology is characterized by severe anemia or the development of cerebral malaria, generally leading to death if left untreated (3). The *Plasmodium* life cycle within its mammalian host includes a non-pathological liver stage followed by red blood cell (RBC) invasion by merozoites, the infectious form of the parasite, which initiates the symptomatic intraerythrocytic cycle (4). Classical malaria paroxysms are characterized by periodic fevers and chills, which are synchronized with the release of merozoites from the infected RBC (iRBC) (5). Furthermore, in the case of *P. falciparum*, the sequestration and destruction of iRBC in the vasculature of lymphoid organs and the brain exacerbates this pathology (6). Disease severity was previously solely attributed to parasite virulence factors (5). However, recent studies have suggested that modulation of the immune system is involved in the development of pathology through the induction of a strong inflammatory response at the beginning of the acute phase, followed by a suppression of the host immune system at later time points (7).

## Malaria and Inflammation

Systemic hyperactivation in *P. falciparum*-infected patients is characterized by elevated levels of circulating nitrogen oxide reactive intermediates (8, 9) and by various cytokines, such as IFNα/γ (10–12), TNFα (5, 13–18), IL-1 (5), IL-6 (19), and the chemokine IL-8 (20). Furthermore, the levels of these cytokines and chemokines have been found to correlate with different manifestations of severe malaria (14, 16–19). Although the generation of these pro-inflammatory molecules favors reduction of the parasitic load, their exacerbated production seems to play a key role in the development of pathology. Both TNFα and IL-1β are considered to be important mediators of fever (3), and participate in the attachment of *P. falciparum*-iRBC to the vascular endothelium (21–23). And *in vivo* studies have demonstrated that the cytokines TNFα and IFNγ are essential for the development of cerebral malaria by inducing the expression of the adhesion molecule ICAM-1 and nitric oxide (NO) (24, 25). Finally, an *in vitro* study has shown that the induction of the pyrogenic molecules MIP-1α and MIP-1β by *Plasmodium* may play an important role in the initiation of fever (26).

During human or murine malaria, phagocytes [e.g., monocytes/macrophages (MØ), and to a lesser extent neutrophils (NØ)] have been demonstrated to play a crucial role in host defense by engulfing free parasites and *Plasmodium*-iRBC, and by eliminating parasites through a respiratory burst-mediated mechanism (27, 28). During the early phase of the infection, the number of phagocytes increases and their activity intensifies (29–33). Moreover, since tissue and circulating MØ are the main source of cytokines during severe malaria (5), it seems that their contribution to the exacerbation of the inflammatory response is also important. For instance, *in vitro* studies have reported the production of several phagocyte-secreted molecules (e.g., IL-1, IL-6, IL-12, and TNFα) (3, 5, 14, 15, 34–40) by human and murine MØ upon contact with *Plasmodium*-iRBC or malarial antigens. Furthermore, the enhanced IFNγ production (19, 41), complement activation (42), and hypergammaglobulinemia observed during the course of acute malarial infection could stimulate cytokine release by MØ (43, 44). Additionally, the production of leukotrienes and reactive oxygen species (ROS) by phagocytes during infection seems to contribute to malaria pathogenesis (5).

## HZ and Malaria

Although enhanced activation of the immune system has been reported during the early stage of the malarial infection, a markedly reduced or absent immune response is observed later during the acute phase of human and murine malaria (45, 46). The most well-studied mechanism explaining this phenomenon is the reduction of T cell proliferation and activity that occurs during malarial infection (47, 48). However, the reduction in T cell numbers is transient (49), and the restoration of their basal numbers does not restore their ability to specifically respond to malaria antigens (29), suggesting that other components of the immune system are also affected. Accordingly, various models of murine malaria have demonstrated that MØ functions (e.g., antigen presentation and microbicidal functions) (7, 10, 29, 50–54) are greatly altered during the course of infection, but the mechanisms involved in the functional modulation of MØ by *Plasmodium* are still incompletely understood. Several lines of evidence suggest that the parasite and its metabolites, principally hemozoin (HZ) and glycosylphosphatidylinositol (GPI), which are released into circulation during the intraerythrocytic cycle, could contribute to the activation and/or the suppression of the immune response (7, 52, 55, 56). The impact of HZ on host physiology and the host response has been a subject of increasingly intensive studies over the last 10 years, and already published data suggest that this metabolite could have an important role in malaria pathophysiology.

Hemozoin is a crystalline, brown pigment that is formed and sequestered in the digestive vacuole of *Plasmodium* as a product of hemoglobin (Hb) catabolism (57). The parasite digests up to 80% of the Hb in the host RBC, which it utilizes as an essential source of nutrients and energy (2). However, this process generates heme, which is highly toxic to the parasites. Since the parasite is unable to excrete the free heme and does not possess a heme oxygenase to recover the iron and detoxify the heme, it aggregates the heme into an insoluble crystal, HZ (58, 59). It was initially thought that this reaction was conducted by a heme polymerase (60). Some proteins have been proposed as candidates (61), but HZ formation does not require the use of a protein or a lipid (62–65), thus this aspect of HZ metabolism remains controversial (61).

*In vivo*, HZ crystals are remarkably uniform in size and shape; however, only certain synthetic protocols allow for the isolation of synthetic crystals with this morphology (58, 66, 67), with many synthetic protocols yielding material that is poorly crystalline (58, 68). HZ is composed of hematin molecules bonded by reciprocal iron–carboxylate linkages to form dimers, which are further connected via hydrogen bonds to form a triclinic crystal (69–71). Although HZ is remarkably thermally stable and insoluble, it has one of the highest concentrations of pro-oxidant sites of any condensed biomaterial, and therefore it may be the source of slowly released oxidation catalysts or the site of surface generated oxidation catalysts. Electron diffraction has been used to index the faces of HZ to determine the specific structures on each surface. The smallest, fastest growing faces are dominated by free propionic acid groups, while the larger faces of the crystal correspond to the hydrophobic flat porphyrin plane of the hematin dimers. Thus, these two pairs of faces on the HZ prisms contrast markedly in nature, with the former being very polar and hydrophilic, and the latter being hydrophobic and lipophilic.

In the past, HZ was only considered to be a metabolic waste, i.e., merely the byproduct of heme detoxification by the parasite (56). However, this molecule has been shown to sequester in various organs (e.g., liver, spleen, and brain), to be actively engulfed by phagocytes, and to modulate MØ functions, indicating that HZ can potentially contribute to the development of malaria immunopathogenesis (2, 26, 72–80). Following the rupture of *Plasmodium*-iRBC, HZ is released from the parasite digestive vacuole and is rapidly engulfed by phagocytes (29, 33, 56). In human and murine malaria, a large number of circulating phagocytes are loaded with HZ, as are phagocytes in the brain and lymphoid organs, such as the spleen (26, 29, 30, 56, 58, 81), where its presence seems to correlate with the severity of the disease. Although HZ is generated during malarial infection caused by all *Plasmodium* species, including the species-infecting mice (e.g., *P. chabaudi* and *P. berghei*), the amount released during severe or cerebral malarial infection due to *P. falciparum*, can be markedly more abundant and localized than compared to mild cases of malaria observed in individuals infected with *P. malariae*, *P. ovale*, or *P. vivax* (82).

## HZ, Immune Cells, and Inflammation

Hemozoin accumulation occurs during erythrocyte rupture: merozoites, along with HZ, free heme, and other contents of the cytoplasm and digestive vacuole of the parasite are released. Many immune cells such as monocytes, macrophages, neutrophils, endothelial cells, and dendritic cells are able to interact with and internalize HZ and iRBC. Among these, the most well-characterized HZ-internalizing cells are the monocytes and macrophages. It has been well documented that human monocytes rapidly engulf HZ, which can fill up to 30% of their total cell volume. Furthermore, the consumed HZ can persist unmodified within the monocytes for long periods of time (83).

Accumulation of HZ in the phagocytic cells of the immune system is used in the diagnosis and prognosis of malaria. In pioneering studies, Laveran described the presence of the pigment granules not only in the iRBC, but also in phagocytes; in some cases, HZ could be detected in RBC that did not contain parasites (84). High levels of HZ within monocytes and neutrophils have been shown to correlate with disease severity. It was observed that adult patients who succumbed to *P. falciparum* infections presented with a higher proportion of HZ-containing neutrophils or monocytes than surviving patients (75). Additionally, it has been shown that children with cerebral malaria have more HZ-containing neutrophils than mildly infected or asymptomatic children (82). Furthermore, patients with severe malaria have iRBC and HZ-laden monocytes in their brain capillaries (85). The same profile of HZ accumulation within the organs has also been observed in the murine model of cerebral malaria (80). The presence of HZ in these immune cells corresponds with its immune modulatory activity.

**Figure 1:**
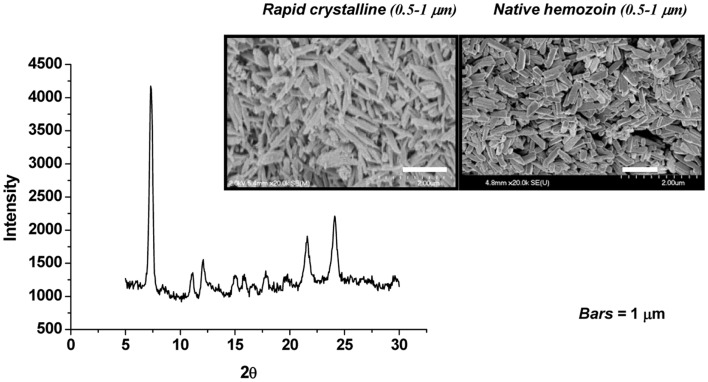
**Synthetic hemozoin analysis**. Scanning electron micrographs of rapid crystalline HZ (rcHZ) and *Plasmodium falciparum*-native HZ. X-ray powder diffraction (XRD) analysis allows the determination of the quality of the crystal. Taken from Ref. (86).

The role of HZ in the modulation of host innate and inflammatory responses has been investigated by many researchers, using different HZ preparation protocols. HZ can be synthesized from hematin (sHZ) or native hemozoin (nHZ) can be purified from iRBC in culture (Figure 1). nHZ needs to be further treated to remove any proteins, lipids, and other materials from disrupted parasite that could have adhered to the highly amphiphilic molecule, in order to obtain a pure product. These HZ preparations have been used to gain a greater and more thorough understanding of the role of HZ in malarial pathology. Although sHZ and nHZ crystals of similar sizes are capable of inducing the same level of inflammatory mediators, sHZ with a smaller or larger crystal size will differently affect the production of pro-inflammatory cytokines. This is believed to occur because the smaller crystal sizes have a greater surface of interaction for a given amount of material (86).

Over the last 10–15 years, several groups have reported that HZ accumulation within MØ modulates several of their functions, and is associated with some malarial symptoms, such as fever, anemia, and splenomegaly (26, 88). It has been determined that human monocytes and murine MØ stimulated with HZ (purified from various species of *Plasmodium* or synthetically generated) produce large amounts of cytokines (IL-10, IL-12, IL-1β, and TNFα), chemokines (MIP-1α and MIP-1β), MIF erythropoietic inhibitor, and adhesion molecules (CD11/CD18) (4, 26, 45, 89). Consistent with these observations, we previously published the first report that *in vivo* inoculation of sHZ rapidly induces the generation of various pro-inflammatory mediators, including myeloid-related proteins (MRPs; S100A8 and S100A9), chemokines (MIP-1α/β, MIP-2, and MCP-1), and cytokines (90); strongly suggesting that HZ itself might play an important role in the development of malaria-related pathologies. Additionally, *in vitro* studies from our laboratory revealed that HZ significantly enhances IFNγ-induced NO production by MØ (91), an important inflammatory event that could favor cerebral malaria development. We also found that native *Pf*HZ- and sHZ-induced MØ chemokine expression is regulated by oxidative stress-dependent (92) and -independent mechanisms. Contrastingly, some *in vitro* studies have shown that MØ which have internalized HZ for a long period of time (over 24 h), exhibited inhibition of PKC (68) and NADPH oxidase (72), IFNγ-inducible class II (MHC-II) expression (45), LPS-induced functions (e.g., CD14, and TNFα) (34, 79, 93, 94), phagocytosis (72), microbicidal activity (74), and the respiratory burst (95). Despite these functional alterations and the possible toxic effects of oxygen radicals and lipoperoxidation triggered following HZ phagocytosis (72, 87, 88), the MØ and monocytes were able to remain viable for several days.

Many studies have reported that HZ induces TNFα gene transcription and expression. TNFα production has been shown to correlate with severe malaria, as it is found in higher concentrations in the serum of patients with severe malaria compared to those with mild malaria (96, 97). Supporting its importance in inflammatory-related processes, HZ-induced TNFα production by human monocytes was found to be inhibited by IgM purified from malaria patients, but not from healthy donors (98). Another important cytokine involved in malarial fever is IL-1β. HZ was found to induce IL-1β expression in an air pouch model (90), and in the liver when intravenously injected (86). Recently, several studies have reported that HZ induces IL-1β production by activating the inflammasome protein complex (99–101). The cellular and molecular mechanisms underlying the ability of HZ to activate the NLRP3/inflammasome complex will be further discussed later in this review. Furthermore, it was also found that HZ can induce the production of IL-6 by endothelial cells *in vitro* and that intraperitoneal administration of HZ can induce IL-6 production *in vivo* (100). Similar findings were also observed in a more controlled *in vivo* environment using an air pouch model (90).

Apart from cytokines, HZ also causes the release of various chemokines and the expression of chemokine receptors, as was briefly mentioned earlier. The engagement of a chemokine with its specific receptor triggers an intracellular signaling cascade that results in chemotactic recruitment of inflammatory cells, leukocyte activation, and antimicrobial effects (102). Using an air pouch model and intravenous injection (90), as well as murine macrophages (86, 103), it was shown that HZ induced the expression of various chemokines (MIP-1α/CCL3, MIP-1β/CCL4, MIP-2/CXCL2, and MCP-1/CCL2) and chemokine receptors (CCR1, CCR2, CCR5, CXCR2, and CXCR4). HZ was also found to augment the production of several β-chemokines in peripheral blood mononuclear cells (PBMC) (104), endothelial cells (100), and *in vivo* (26, 105). These results strongly support the role of HZ as a potent pro-inflammatory agent that could contribute to the immunopathology of malaria observed in humans and murine malaria.

## HZ/Phagocyte Interaction: From Basic Signaling to NLRP3 Inflammasome

As mentioned above, HZ is capable of activating different cell types resulting in the release of several pro-inflammatory and anti-inflammatory mediators. However, the intracellular mechanisms underlying HZ-induced cellular events are still under investigation. An initial study revealed a synergism between HZ and IFNγ resulting in the induction of NO production. The generation of the microbicidal agent required the activation of extracellular signal-regulated kinase (ERK) 1/2 pathway, but was independent of an IFNγ-induced activation of the JAK2/STAT-1 pathway (91). However, both ERK and JAK2/STAT-1 signaling was found to be necessary to attain maximal NF-κB activation and iNOS promoter-binding capability (91). NF-κB was also shown to be greatly involved in HZ-induced chemokine expression (103). In addition to MAP kinases, HZ has recently been described to be capable of activating spleen tyrosine kinase (Syk), augmenting inflammasome activation and IL-1β production by THP-1 human monocytic cells and murine macrophages (99). In the same study, it was revealed that kinases downstream of Lyn/Syk, for instance, MAP kinase family members, might be involved in inflammasome activation, since inhibition of ERK, but not p38, decreased IL-1β production.

**Figure 2:**
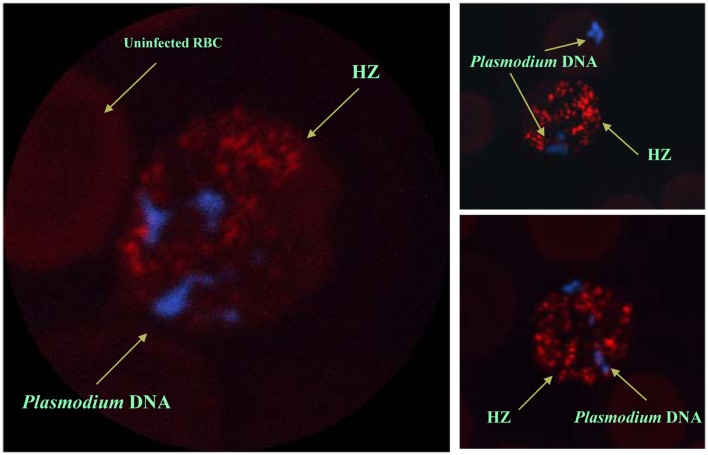
***In situ* localization of *Plasmodium* hemozoin and parasitic DNA**. Confocal pictures of RBC from *Plasmodium chabaudi* DK-infected mice. Selected images of schizonts and late trophozoites stages of iRBC. DAPI staining was used to visualize malarial DNA (blue). No staining was required to localize HZ since it autofluorescences (red). Even after merozoite release from the iRBC (see top right panel), malarial DNA was never in contact with hemozoin. Images were taken from Ref. (86).

Despite the fact that HZ has been shown to be immunologically active *in vitro* and *in vivo*, the cellular receptors recognizing HZ remain elusive. However, the efficiency of HZ-induced signaling and phagocyte function seem to depend on its internalization and lipid raft integrity. It is well known that the cells of the innate immune system recognize pathogen-associated molecular patterns (PAMPs) or damage-associated molecular patterns (DAMPs) by expressing gene-encoded pattern recognition receptors (PRR), such as Toll-like receptors (TLRs), Nod-like receptors (NLRs), c-type lectin, and RIG-like helicases. TLRs can recognize *P. falciparum* GPI anchors (106), but the HZ-induced response is not dependent on TLRs (86, 100, 107, 108). Nevertheless, the ability of TLRs to sense HZ is still a controversial issue. It is important to be fastidious in the interpretation of these results, as the amphiphilic nature of HZ allows it to bind certain proteins, lipids, and nucleic acids; therefore, any *Plasmodium* molecules adhering to HZ during the preparation of nHZ could be sensed by the TLRs. In this context, Parroche et al. (107) proposed that HZ was a carrier of a TLR ligand and that the immune response induced by HZ was from a possible contamination of HZ with *Plasmodium* DNA. However, different research groups have shown that synthetic and native HZ that are not contaminated by DNA (86, 109) are still very powerful immunogenic molecules (83). Furthermore, by using DNA staining and the natural red/green fluorescence of HZ, it was shown that *Plasmodium* DNA within iRBC never co-localizes with HZ, which is confined to the food vacuole (Figure 2) (86). In this regard, the ability of TLRs to recognize HZ is still unclear, as is the ability of other PRRs to recognize HZ, especially NLRs.

The NLR family of receptors is characterized by three domains: a leucine-rich repeat (LRR) domain in the N-terminus, a central nucleotide-binding domain, and a variable C-terminus. Based on the composition of the C-terminus and central domain, NLRs are divided into different subfamilies: the *NLRB subfamily* (which consists of one member, NAIP), the *NLRC subfamily* (NLRC4/IPAF and NOD1, which are NLR containing a CARD domain, and NOD2, which contains two CARD domains); and the *NLRP subfamily* (NLRP1 and NLRP3, NLR containing a pyrin domain). The members of each subfamily recognize different pathogen-associated molecules; for example: flagellin is recognized by NLRC4, anthrax lethal toxin by NLRP1, muramyl dipeptide and lysin-peptidoglycan by NOD2, meso-diaminopimelic acid-peptidoglycan by NOD1, and a vast spectrum of ligands (bacterial RNA, inorganic materials, gout-associated crystal-MSU, and microbes) by NLRP3. NOD1/NOD2 receptor stimulation has been shown to induce RIP2 kinase-dependent NF-κB activation, resulting in the transcription of pro-inflammatory cytokines (110–112). Recent findings suggest that certain pro-inflammatory events occurring during *P. berghei* ANKA infection may depend on NOD2 (113), however the role of HZ in this circumstance is still incompletely understood. Furthermore, several laboratories have made observations indicating that the NLRP3 inflammasome complex could be involved in a HZ-induced response.

**Figure 3:**
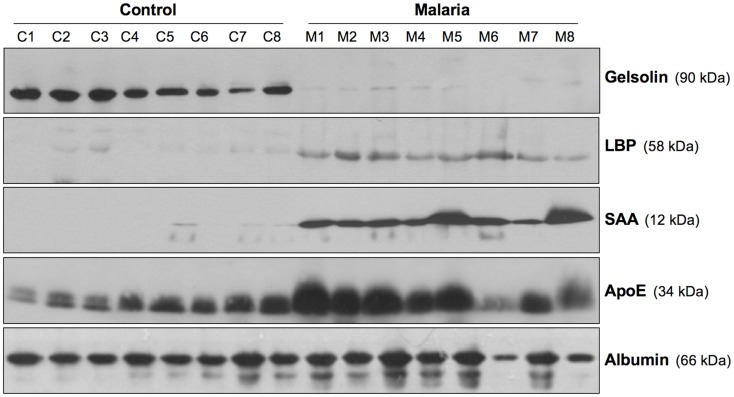
**Inflammatory biomarkers adhering to hemozoin**. Sera biomarkers from malaria patients and healthy individuals were detected using Western blotting. The membranes were blotted using antibodies specific for gelsolin, LPS-binding protein (LBP), serum amyloid A (SAA), apolipoprotein E (ApoE), and serum albumin. C1–C8, control; M1–M8, malaria. Figure was taken from the supplemental data of Ref. (116).

The potential role of the NLRP3 inflammasome in HZ-triggered inflammatory events is of particular interest, because IL-1β is known to be integral to the fever episodes and pro-inflammatory processes observed during *Plasmodium* infection. However, the results regarding the participation of the inflammasome complex have been slightly inconsistent. Studies by our laboratory (99) and by Dostert et al. (101) showed that HZ-induced IL-1β production and neutrophil recruitment were dependent on the NLRP3 inflammasome. In partial agreement, Griffith et al. (100) showed that HZ-stimulated neutrophil recruitment into the mouse peritoneal cavity was dependent on NLRP3 inflammasome. Additionally, using a murine model, three different studies have demonstrated that NLRP3-deficient mice showed some level of protection against two different murine parasites, *P. berghei* ANKA and *P. chabaudi adami* DS (99, 101, 114). Nevertheless, this protection cannot be solely attributed to HZ, as during the course of *Plasmodium* infection, a number of factors from the pathogen and the host immune system will contribute to the outcome of the infection. Moreover, our study revealed that *Plasmodium*-infected NLRP3- and IL-1β-deficient mice have a lower body temperature compared to wild-type. This finding is consistent with the potential role of HZ during malarial infection, as it is released during iRBC lysis, which is usually followed by an episode of fever. Furthermore, the laboratory of Scharzwer recently reported that the attachment of fibrinogen to HZ imbued HZ with a greater capacity to activate host inflammatory functions (115). In this context, recent data from our laboratory (116) revealed that HZ interacts with a large number of inflammatory-related biomarkers (e.g., fibrinogen, serum amyloid A (SAA), LPS-binding protein (LBP), and apolipoproteins) found in the circulation of *P. falciparum*-infected patients (Figure 3). The potential of these molecules to modify the interaction of HZ with immune cells is of great interest, as it could exacerbate the inflammatory events occurring during malaria.

**Figure 4:**
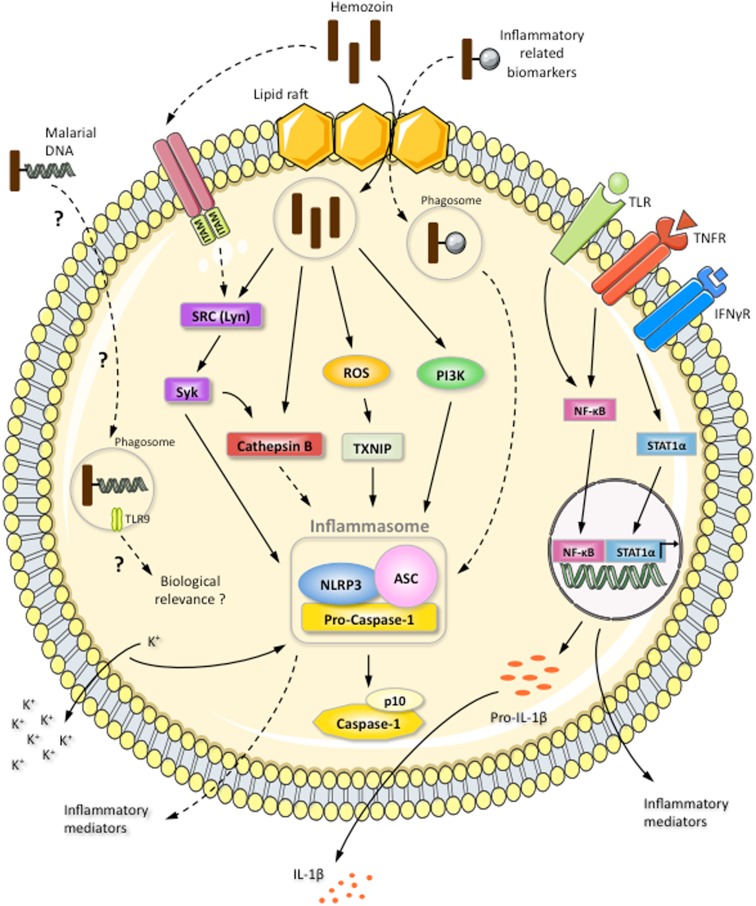
**Induction of NLRP3 inflammasome complex by the inorganic crystal HZ**. HZ induces IL-1β production via the NLRP3/ASC inflammasome: activation of caspase-1 results in the cleavage of pro-IL-1β. Pro-IL-1β expression is resultant of the TLR4- and TNFα-activated NF-κB pathway or the IFNγ-stimulated STAT-1α pathway. The HZ-activated NLRP3 inflammasome is dependent on potassium efflux, ROS generation, HZ internalization, and cathepsin B activation. HZ internalization and the induction of SRC kinase signaling are mediated by lipid rafts. An ITAM-containing receptor could also be the starting point of SRC kinase cascade. HZ is internalized within a phagosome-like vesicle surrounded by LAMP-1. HZ activation of the SRC kinase Lyn leads to Syk phosphorylation. Subsequently, Syk positively modulates cathepsin B activation, which could result in the induction of the NLRP3 inflammasome. HZ is also capable of activating the NLRP3 inflammasome through PI3 kinase. The involvement of malarial DNA, which can adhere to HZ, in the activation of an intracellular receptor and its biological relevance are controversial and only reported for dendritic cells, which are present in limited numbers in the blood. HZ can interact with a large number of inflammatory-related biomarkers found in the circulation of *P. falciparum*-infected patients. However, the effect of these biomarkers on NLRP3 inflammasome activation is still unknown. Continuous arrows indicate a positive modulation. Dotted arrows indicate a hypothetical effect. Dotted arrows with a question mark indicate an unknown or controversial effect.

The mechanisms underlying the activation of the NLRP3/inflammasome complex by HZ are currently under investigation. Three models have been proposed: potassium channel efflux, lysosome rupture, and/or ROS generation (117) (Figure 4). Two independent groups have illustrated the involvement of potassium efflux, phagocytosis, and ROS generation in inflammasome activation (99, 101). However, there is a discrepancy in whether cathepsin B is involved, depending on the approach used. Cathepsin B-deficient mice showed no effect (101), whereas cathepsin B-specific inhibitors were found to block inflammasome activation (99). Nevertheless, HZ-triggered inflammasome activation seems to involve at least two of the proposed models (potassium channel efflux and ROS generation) and cathepsin B activation. Disruption of the phagolysosome by HZ does not appear to occur, since HZ has been shown to be contained in vacuoles surrounded by LAMP-1, and cathepsin B has not been found in culture supernatant, which is generally the case for asbestos and silica (99), which not only disrupt the phagolysosome, but also kill the phagocytic cells. Of utmost importance, our study revealed that Lyn/Syk kinases are the upstream signaling partners in the activation of the NLRP3/inflammasome complex (Figure 4). The participation of these kinases in HZ-induced inflammasome activation is highly suggestive that an ITAM-containing receptor on the surface of the host cell could be the starting point for this biochemical cascade. However, it is also possible that HZ is capable of modulating the lipid raft environment, which could initiate the signaling cascade (Figure 4).

Finally, it is important to stress that inorganic crystals like asbestos, silica, and MSU not only disrupt phagolysosome integrity, but are also highly apoptotic and disruptive to cell integrity. Conversely, HZ is able to stay within host cells for long periods of time, from several days to weeks, without discernably affecting phagocyte viability (118). Moreover, HZ is markedly smaller than the other inorganic crystals mentioned above and is fully engulfed by the host cells, unlike the other crystals mentioned.

In conclusion, it is has been established that HZ is a powerful modulator of the innate immune response, which suggests that it has the potential to be detrimental or beneficial to the host depending on the stage of the infection. Furthermore, it has recently been demonstrated that HZ is sensed as a danger signal, resulting in the activation of the inflammasome (99, 101). However, there are contradicting results regarding the modulation of the immune response by HZ. These differences can be explained at least in part by the different cell types and incubation times used in various studies, and most prominently, by the quality of the HZ crystal utilized. Therefore, a unified method to generate sHZ crystals, which more closely resembles the ones naturally produced by *Plasmodium*, needs to be established. Additionally, ensuring that the resulting sHZ crystals possess the correct quality, size, and crystallinity by using X-ray powder diffraction (XRD) analysis would aid in attaining more reproducible data.

## Conflict of Interest Statement

The authors declare that the research was conducted in the absence of any commercial or financial relationships that could be construed as a potential conflict of interest. The editor declares that while the authors of the manuscript and himself are currently employed by McGill University, and that the author recently published a paper alongside one of the co-authors, there has been no conflict of interest during the review and handling of this manuscript.
